# Photobiomodulation in chronic pain: a systematic review of randomized clinical trials

**DOI:** 10.3389/fnint.2026.1717372

**Published:** 2026-02-03

**Authors:** Luciano Maia Alves Ferreira, Ana Beatriz Cabral Oliveira, Jose João Baltazar Mendes, Gabrielle Vitoria Costa, Izadora Reis Silva, Gabrielly Nogueira Santos, Gabrielly Santos Pereira, Marcelo Lourenço Silva

**Affiliations:** 1 Neuromodulation and Pain Lab (NeuroPain), Egas Moniz Interdisciplinary Research Center (CiiEM), Almada, Portugal; 2 Laboratory of Neuroscience, Neuromodulation and Study of Pain (LANNED), Federal University of Alfenas (UNIFAL-MG), Alfenas, Brazil

**Keywords:** chronic pain, efficacy, photobiomodulation, randomized controlled trial, safety, systematic review

## Abstract

**Introduction:**

Photobiomodulation (PBM) stands out as a promising therapeutic alternative for the management of chronic pain, but there is still controversy regarding its efficacy and safety, given the diversity of protocols and populations evaluated.

**Objectives:**

To critically review the available literature on the use of PBM in adults with chronic pain conditions, synthesizing the evidence on analgesic and functional effects, impact on quality of life, and safety profile. Methods: A systematic search was conducted in PubMed, Embase, Scopus, LILACS, and MEDLINE, including articles published between September 2015 and September 2025. Randomized clinical trials that compared PBM protocols to placebo, sham, or conventional care were selected. The outcomes investigated included pain intensity (primary), function, quality of life, and occurrence of adverse events (secondary).

**Results:**

Fourteen studies were included, covering populations with fibromyalgia, peripheral neuropathies, orofacial pain, and musculoskeletal pain. Most trials demonstrated significant pain reduction with PBM, particularly in fibromyalgia and neuropathy. In some studies, functional gains and improved quality of life were observed. The incidence of adverse events was low, reinforcing the method’s safety, although the heterogeneity of technical parameters compromises the standardization of results.

**Conclusion:**

PBM has analgesic potential and a safe profile for managing chronic pain, especially in cases difficult to control with conventional therapies. However, the variability of clinical parameters and limited follow-up still hinder more comprehensive recommendations. Additional multicenter studies with standardized protocols are needed to consolidate clinical guidelines.

**Systematic review registration:**

https://www.crd.york.ac.uk/PROSPERO/view/CRD420251140711, Identifier: CRD420251140711.

## Introduction

Chronic pain is a complex condition that persists beyond the expected time of tissue healing or arises from long-standing pathological processes that generate continuous or recurrent pain (Treede et al., 2015). It affects a substantial portion of the global population. Current estimates suggest that 20% of adults experience pain at some point in life, while about 10% are diagnosed with chronic pain each year, totaling nearly 60 million people worldwide (Goldberg and McGee, 2011).

Several risk factors contribute to chronic pain, including advanced age, female sex, sleep disturbances, mood disorders, obesity, fatigue, and neurocognitive impairments. Psychiatric comorbidities such as depression and anxiety are common and exacerbate the reduction in quality of life, while also increasing the risk of inappropriate opioid use and reliance on invasive therapie (GBD 2019 Risk Factors Collaborators, 2020).

According to the International Classification of Diseases (ICD-11), chronic primary pain is recognized as a disease in its own right, distinct from chronic secondary pain, which develops as a consequence of specific conditions such as visceral disease, neuropathies, musculoskeletal disorders, cancer, surgery, trauma, and headaches, including orofacial pain (Treede et al., 2015). From a pathophysiological perspective, chronic primary pain involves complex alterations in nociceptive modulation at both peripheral and central levels. These include nociceptor sensitization in peripheral terminals and dysfunction of ascending and descending pain pathways (Dubin and Patapoutian, 2010; Baron et al., 2017). Such processes sustain pain perception even in the absence of active tissue injury, characterizing nociplastic pain (Kosek et al., 2016). This understanding underscores the need for therapies that modulate central and peripheral mechanisms, such as photobiomodulation (PBM) (Navarro-Ledesma et al., 2024).

PBM applies red or near-infrared light to stimulate cellular responses, reduce inflammation, modulate nociceptive activity, and promote tissue repair. Preclinical and clinical studies suggest that PBM is a promising strategy for chronic pain management, although variability in protocols and outcome measures has limited the consolidation of consistent evidence (Chow et al., 2009; Furquim et al., 2023).

Mechanistically, PBM acts on both peripheral and central nociception. In tissues, PBM stimulates mitochondrial activity, enhancing ATP production and supporting cell repair (Hamblin, 2017). It also reduces the release of inflammatory mediators such as prostaglandins and proinflammatory cytokines (IL-1β, IL-6, TNF-*α*), thereby decreasing edema and nociceptor sensitization (Chung et al., 2012). These anti-inflammatory effects are complemented by improved microcirculation and lymphatic flow, which enhance oxygenation and accelerate the removal of algogenic metabolites (Bjordal et al., 2006).

At the peripheral level, PBM decreases the excitability of Aδ and C fibers, while centrally it modulates spinal and supraspinal pain pathways (Chow et al., 2009; Chung et al., 2012). PBM also promotes the release of inhibitory neuromodulators such as serotonin and acetylcholine, which contribute to reducing both peripheral and central sensitization (Gigo-Benato et al., 2004; Leal-Junior et al., 2020). Together, these mechanisms mitigate characteristic symptoms of chronic pain, such as allodynia and hyperalgesia, and may enhance muscle and joint function. Clinically, PBM has demonstrated potential to reduce pain intensity, lower the need for analgesics and opioids, and improve patients’ quality of life (Hamblin, 2017; Leal-Junior et al., 2019).

In this context, the present review aims to critically analyze the literature on photobiomodulation for chronic pain, focusing on its analgesic, functional, and quality-of-life effects, as well as safety and adverse events.

## Methods

### Study design

This systematic review evaluates the effects of PBM on chronic pain conditions in adults. The review followed the Preferred Reporting Items for Systematic Reviews and Meta-Analyses (PRISMA) 2020 guidelines and was prospectively registered in PROSPERO (CRD420251140711).

### Eligibility criteria

The eligibility criteria were defined using the PICO framework:

Population: Adults (≥18 years) diagnosed with chronic pain conditions, including fibromyalgia, headache disorders, temporomandibular disorders, neuropathic pain (e.g., chemotherapy-induced, diabetic, or leprosy-related), chronic neck/shoulder pain, or post-COVID-19 orofacial pain. Studies had to explicitly report pain duration of at least three months. Intervention: Trials employing photobiomodulation (PBM), delivered via laser or light-emitting diode (LED) devices, with therapeutic intent to reduce pain or improve function. Interventions were required to describe stimulation parameters, including wavelength, energy dose, treatment duration, frequency, and total number of sessions. Comparison: Sham or placebo photobiomodulation, no intervention, conventional care, or alternative stimulation protocols. Outcomes: The primary outcome was pain reduction, measured by validated tools such as the VAS (VAS), Numeric Rating Scale (NRS), Brief Pain Inventory (BPI), Fibromyalgia Impact Questionnaire (FIQ/FIQR), or neuropathic pain instruments (e.g., LANSS, Pain DETECT, DN4, Modified Total Neuropathy Score). Secondary outcomes included functional capacity (e.g., Six-Minute Walk Test, Timed Up and Go, jaw function), quality of life (e.g., SF-36, quality of life, VAS), psychological variables (e.g., anxiety, depression, catastrophizing, kinesiophobia), and adverse events. Study design: Only randomized controlled trials (RCTs) published in peer-reviewed journals were included. Non-randomized studies, case reports, reviews, editorials, and conference abstracts were excluded from the analysis.

### Information sources

Searches were conducted in the following electronic databases: PubMed, Embase, Scopus, LILACS, and MEDLINE via BVS. The search covered publications from September 1, 2015 to September 1, 2025, and included articles in English, Spanish, or Portuguese. Additional studies were identified through manual search of reference lists and consultation with field expert.

### Search strategy

The search strategy was constructed using a combination of MeSH terms and free-text keywords related to photobiomodulation and chronic pain. Boolean operators AND and OR were applied as follows:

(“Photobiomodulation” OR “Low-Level Light Therapy” OR “Near-Infrared Light” OR “Photobiomodulation Therapy”)

AND (“Chronic Pain” OR “Fibromyalgia” OR “Headache” OR “Temporomandibular Disorders” OR “Neuropathic Pain” OR “Neck Pain” OR “Orofacial Pain”).

AND (“Randomized Controlled Trial” OR “Sham” OR “Placebo” OR “Clinical Trial”).

Full search strategies for each database (e.g., PubMed, Embase, Scopus, Web of Science, Cochrane Library) are available in the PROSPERO record.

### Study selection

All identified studies were imported into Rayyan for duplicate removal and screening. Two independent reviewers (GSP and MLS) screened titles and abstracts, followed by full-text analysis based on the inclusion criteria. Disagreements were resolved through discussion or by a third reviewer (LMAF). The selection process was documented using a PRISMA flow diagram.

### Data extraction

Data were independently extracted by two reviewers using a standardized form. Extracted information included:

Study characteristics: author, year of publication, country, design, and sample size.

Population: diagnosis of chronic pain condition, age, and sex distribution.

Intervention: transcranial photobiomodulation parameters, including device type, wavelength, energy dose, treatment duration, frequency, and total number of sessions.

Comparator: sham/placebo photobiomodulation, no intervention, standard care, or alternative stimulation protocols.

Outcomes: pain intensity (type and timing of measurement tools), functional assessments, quality of life instruments, psychological measures, and reported adverse events.

When relevant data were missing, attempts were made to contact study authors directly.

### Risk of bias assessment

**Figure 1:**
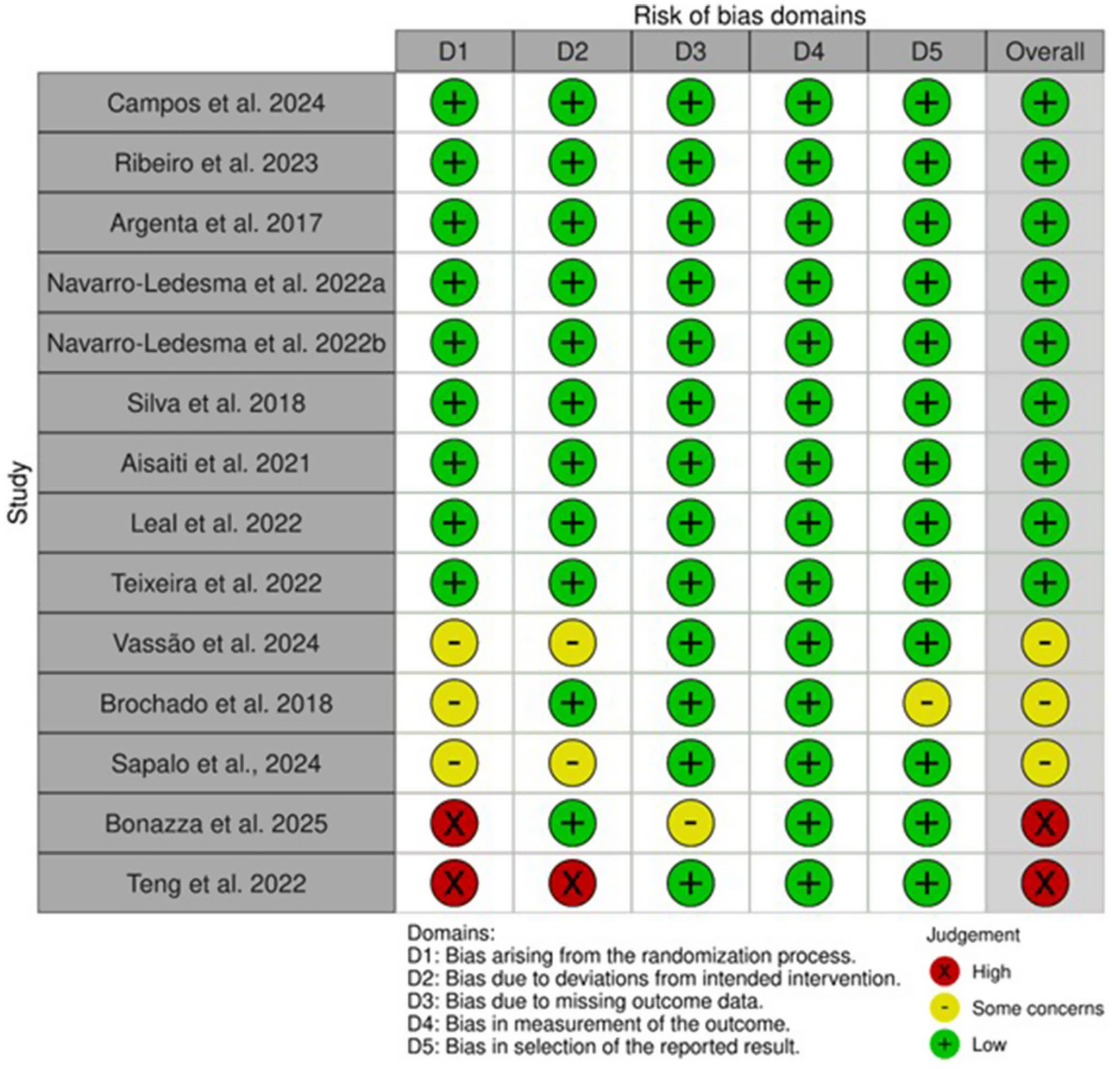
Risk of bias assessment.

The RoB 2.0 tool (Cochrane Collaboration) was used to assess risk of bias in RCTs across five domains: randomization process, deviations from intended interventions, missing outcome data, measurement of outcomes, and selection of the reported result. Each domain was rated as “low risk,” “some concerns,” or “high risk.” The overall risk of bias assessment of the included randomized controlled trials is summarized in Figure 1.

### Data synthesis

A narrative synthesis was conducted for all included studies, structured to compare population characteristics, PBM intervention protocols (including wavelength, energy dose, frequency, duration, and treatment sites), comparator groups, and reported outcomes. The synthesis focused primarily on pain outcomes, assessed through validated scales such as the VAS (VAS), Numeric Rating Scale (NRS/NPRS), Brief Pain Inventory (BPI), Fibromyalgia Impact Questionnaire (FIQ/FIQR), and neuropathic pain instruments (LANSS, PainDETECT, DN4, Modified Total Neuropathy Score). Secondary outcomes included functional capacity, quality of life, psychological measures (e.g., anxiety, depression, catastrophizing, kinesiophobia), and safety (adverse events, dropouts).

Studies were grouped by clinical condition (e.g., fibromyalgia, temporomandibular disorders, neuropathic pain, chronic neck/shoulder pain, post-COVID-19 orofacial pain) to enable condition-specific comparisons. Patterns were identified regarding protocol parameters (e.g., low vs. high dose, localized vs. whole-body application, wavelength ranges) and their effects on pain relief, functional recovery, and quality of life. Attention was given to whether outcomes were measured immediately post-intervention or at follow-up, and to the duration and clinical significance of observed effects.

Due to heterogeneity in intervention protocols, outcome measures, and reporting formats, a quantitative meta-analysis was not feasible. Instead, findings were synthesized descriptively to highlight consistent trends, protocol-specific effects, and current gaps in the literature, including the need for standardized reporting of stimulation parameters and long-term follow-up data.

## Results

**Figure 2:**
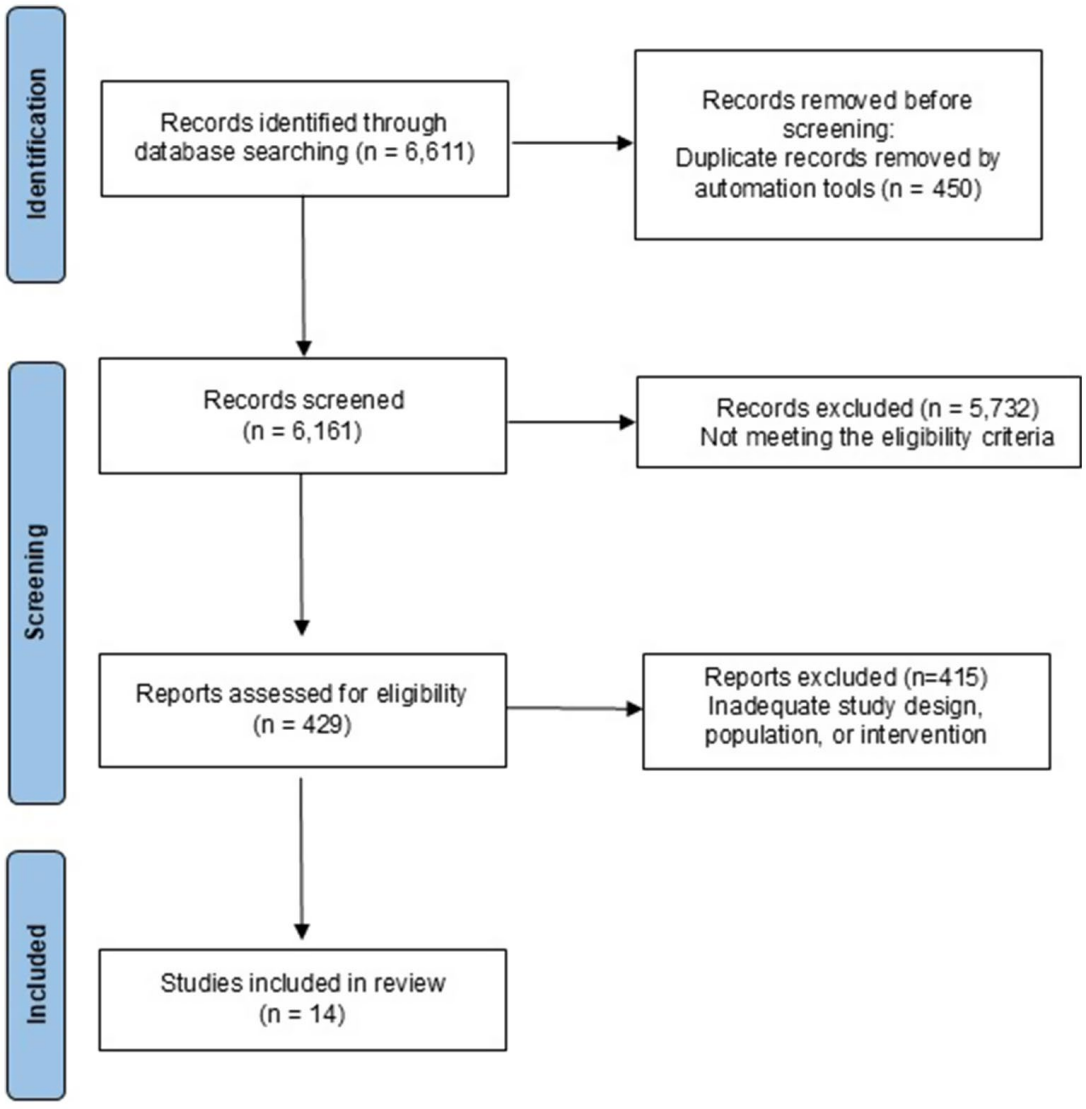
PRISMA flow diagram.

A systematic search in PubMed, Web of Science, ScienceDirect, and Scopus databases yielded 6,611 studies. After removing 450 duplicates, 6,161 articles were screened as potentially eligible for inclusion. Following title and abstract screening, 5,732 records were excluded for not meeting the eligibility criteria. A total of 429 full-text articles were assessed for eligibility, of which 415 were excluded for reasons such as inadequate study design, population, or intervention. Ultimately, 14 studies met all inclusion criteria and were included in the systematic review. The PRISMA flow diagram summarizing this selection process is presented in Figure 2 (see Table 1).

**Table 1:** Summary of randomized controlled trials investigating photobiomodulation in chronic pain.

Study	Study design	Population	Intervention protocol	Comparison group	Pain measure	Treatment effect	Statistical significance
Campos et al. (2024)	Double-blinded randomized controlled trial	Post-COVID-19 orofacial pain and tension-type headache	ECCO Reability device, 660 ± 10 nm, 100 mW, 30 min/week × 4 weeks, left radial artery	Sham PBM (inactive device)	Visual analog scale, brief pain inventory	Significant reduction in pain (visual analog scale, brief pain inventory) in VAScular PBM versus sham; no difference in headache impact test-6	p = 0.010 (visual analog scale), *p* = 0.009–0.016 (brief pain inventory)
Ribeiro et al. (2023)	Triple-blinded randomized controlled trial	Fibromyalgia	PBM with static magnetic field, 9 sessions (3/week × 3 weeks)	Placebo PBM with static magnetic field	Tender point count	Decrease in tender points in PBM with static magnetic field versus placebo	p < 0.0001
Teng et al. (2022)	Single-blinded, sham-controlled randomized controlled trial	Chemotherapy-induced peripheral neuropathy	Laser PBM, 2/week × 6 weeks	Sham control	Chemotherapy-induced peripheral neuropathy response	48% (PBM) versus 53% (control) at 6 weeks; 45% versus 33% at 12 weeks; improvement in both, sustained in PBM	p < 0.001
VASsão et al. (2024)	Double-blinded randomized controlled trial	Fibromyalgia	PBM (Antares, cluster probe), 20/32/40 J, 2/week × 12 weeks, various muscles	No intervention (control group); Placebo PBM plus exercise (exercise plus placebo PBM group)	VAS	Significant within-group pain reduction in all treated groups; no intergroup difference	*p* < 0.05 (intragroup)
Argenta et al. (2017)	Double-blinded, sham-controlled, cross-over trial	Chemotherapy-induced peripheral neuropathy	PBM with or without physiotherapy, 30 min, 3/week × 6 weeks	Sham PBM	Modified total neuropathy score	Significant reduction in modified total neuropathy score in PBM versus sham	p < 0.001
Navarro-Ledesma et al. (2022)	Triple-blinded randomized controlled trial	Fibromyalgia	NovoTHOR whole-body PBM, 660/850 nm, 25.2 J/cm^2^, 20 min, 3/week × 4 weeks	Sham PBM (placebo mode)	Numeric pain rating scale	Significant between-group reductions (NPRS) at 4 weeks (post-treatment, T2) and 2-week follow-up (T3)	*p* < 0.001
da Silva et al. (2018)	Double-blinded randomized controlled trial	Fibromyalgia	Cluster (905/640/875 nm), 39.3 J/location	No mention found	Visual analog scale, fibromyalgia impact questionnaire	Improved pain threshold (PPT), pain perception (VAS), FIQ and reduced tender points in PBM	p < 0.05
Brochado et al. (2018)	Single-blinded randomized controlled trial	Temporomandibular disorder (myogenic/arthrogenic)	Gallium-aluminum-arsenide diode laser, 808 nm, 100 mW, 133 J/cm^2^, 3/week × 4 weeks	No placebo group; active comparators only	VAS	Pain reduction in all groups (PBM, manual therapy, conventional therapy)	*p* < 0.001 (within-group)
Aisaiti et al. (2021)	Double-blinded randomized controlled trial	Temporomandibular disorder (myalgia/arthralgia)	Gallium-aluminum-arsenide laser, 810 nm, 100 mW, 6 J/cm^2^, 1/day × 7 days	Sham PBM (inactive laser)	Numeric rating scale	Greater pain reduction in PBM versus placebo (arthralgia); no difference (myalgia)	*p* = 0.014 (arthralgia)
Teixeira et al. (2022)	Triple-blinded randomized controlled trial	Chronic neck/shoulder pain	MR4 Photobiomodulation, 230.85 J/session, 9 min, 2/week × 3 weeks	Placebo PBM with static magnetic field (inactive device)	VAS (0–100)	Lower pain in PBM with static magnetic field versus placebo at all timepoints	*p* < 0.05
Leal et al. (2022)	Double-blinded randomized controlled trial	Diabetic neuropathy	Laser PBM, 808 nm, 12 J/cm^2^, 3/week × 30 days	Standard care (medication); Placebo PBM	Visual analog scale, Leeds assessment of neuropathic symptoms and signs, PainDETECT	Significant pain reduction in PBM versus control/placebo	*p* < 0.05
Bonazza et al. (2025)	Double-blinded randomized controlled trial	Leprosy neuropathic pain	Pulse laser (Ibramed I42), 904 nm, 70 mW, 4 J/cm^2^, 3/week × 4 weeks	Sham PBM (device off) plus physiotherapy	VAS	Pain reduction in both groups; no intergroup difference	*p* = 0.041 (PBM plus physiotherapy), *p* = 0.0061 (physiotherapy)
André Timóteo et al. (2024)	Double-blinded randomized controlled trial	Diabetic neuropathy	Light-emitting diode PBM, 3 days	Placebo	Douleur Neuropathique 4	Significant reductions (4.7–5.5 points on DN4) in all PBM groups (Red, Infrared, Red+Infrared) compared with control and sham.	*p* < 0.001 for LED vs. control and LED vs. sham

### Study selection and characteristics

From an initial pool of 6,611 articles, 14 studies met the eligibility criteria and were included in this review. The included trials encompassed diverse chronic pain populations: five studies in fibromyalgia (Navarro-Ledesma et al., 2024; Ribeiro et al., 2023; Vassão et al., 2024; Navarro-Ledesma et al., 2022; da Silva et al., 2018), two in temporomandibular disorders (TMD) (Brochado et al., 2018; Aisaiti et al., 2021), two in chemotherapy-induced peripheral neuropathy (Teng et al., 2022; Argenta et al., 2017), two in diabetic neuropathy (Leal et al., 2022; André Timóteo et al., 2024), and one study each in post-COVID-19 orofacial pain with tension-type headache (Campos et al., 2024), chronic neck/shoulder pain (Teixeira et al., 2022), and neuropathic pain related to leprosy (Bonazza et al., 2025).

Interventions varied considerably, with protocols including laser-based photobiomodulation (GaAlAs, Antares, MR4, Ibramed), NovoTHOR whole-body photobiomodulation, cluster diode devices, ECCO Reability, and LED systems. Wavelengths ranged from 660 to 905 nm, session frequency from once weekly to daily, and treatment duration from 3 days to 12 weeks. Comparators included sham or placebo photobiomodulation in eight studies, no intervention in one, standard care (medication) in one, exercise/manual therapy as active comparators in two, and placebo + exercise in one.

### Primary outcomes: pain intensity

Campos et al. (2024) reported significant reductions in VAS and BPI scores with VAScular PBM compared to sham (*p* = 0.010). Ribeiro et al. (2023) found a marked decrease in tender point counts in fibromyalgia patients (*p* < 0.0001). Argenta et al. (2017) observed a 32–53% reduction in mTNS in chemotherapy-induced neuropathy (*p* < 0.001). Teng et al. (2022) reported sustained improvement in CIPN response rates in the PBM group compared to controls (45–48% vs. 33–53%, *p* < 0.001). Other trials showed within-group improvements without significant between-group differences (Vassão et al., 2024; Bonazza et al., 2025).

### Secondary outcomes: functional and quality of life measures

Campos et al. (2024) reported improvements in BPI functional domains, including walking, work, sleep, and enjoyment. da Silva et al. (2018) observed significant gains in quality of life and function with PBM and exercise, particularly when combined. Navarro-Ledesma et al. (2022) found improvements in health-related quality of life (VAS HRQL) following whole-body PBM. Brochado et al. (2018) demonstrated improved jaw function and reductions in anxiety/depression across PBM, manual therapy, and combined groups.

In contrast, Teixeira et al. (2022) reported no significant difference in functional outcomes between PBM and placebo.

### Risk of bias

Nine studies were judged to have a low risk of bias across all domains (Navarro-Ledesma et al., 2024; Campos et al., 2024; Ribeiro et al., 2023; Argenta et al., 2017; Navarro-Ledesma et al., 2022; da Silva et al., 2018; Aisaiti et al., 2021; Teixeira et al., 2022; Leal et al., 2022). Three studies were classified as having some concerns regarding overall risk of bias (Vassão et al., 2024; Brochado et al., 2018; André Timóteo et al., 2024), while two studies were assessed as presenting a high risk of bias (Teng et al., 2022; Bonazza et al., 2025).

Across domains, most trials demonstrated a low risk of bias related to outcome measurement and selection of reported results, indicating adequate assessor blinding and appropriate reporting of prespecified outcomes. In contrast, the randomization process was the most frequent source of concern, as several studies failed to clearly report allocation concealment procedures or methods of random sequence generation (Teng et al., 2022; Brochado et al., 2018).

Concerns related to deviations from intended interventions were observed in the study by Vassão et al. (2024), mainly due to insufficient reporting of adherence monitoring and protocol fidelity. Regarding missing outcome data, most trials adequately addressed attrition; however, Bonazza et al. (2025) did not provide sufficient information concerning participant dropouts.

Overall, although the majority of studies exhibited a low risk of bias, limitations related to incomplete reporting of randomization procedures and intervention adherence were identified, which may affect the internal validity of some trials evaluating photobiomodulation for chronic pain.

### Safety and adverse events

Safety profiles were consistently favorable. Thirteen of the 14 trials reported no adverse events, while Teng et al. (2022) described only low-grade side effects in both active and control groups. These low-grade side effects consisted primarily of transient local warmth, mild discomfort at the application site, and temporary tingling sensations, all of which resolved spontaneously without the need for intervention. No serious adverse events or intervention-related dropouts were reported in any study.

### Limitations

Despite promising findings, heterogeneity in devices, wavelengths, doses, and outcome measures complicates cross-trial comparison. Many studies had small sample sizes, limited follow-up, and incomplete reporting of intervention parameters or adverse events, reducing the generalizability of results. In addition to study-level limitations, this systematic review has inherent limitations that should be acknowledged. The restriction to published randomized controlled trials may have introduced publication bias, as studies with null or negative findings are less likely to be published. Furthermore, the substantial methodological heterogeneity across trials precluded the performance of a meta-analysis, limiting quantitative synthesis of effect sizes. Variability in PBM protocols, outcome measures, and follow-up durations also constrained direct comparison across studies and reduced the ability to draw definitive conclusions regarding optimal treatment parameters.

## Discussion

This review compiles and critically examines findings from fourteen RCTs that explored the application of PBM in various chronic pain conditions. These include fibromyalgia, peripheral neuropathies, musculoskeletal disorders, and orofacial pain following COVID-19. The collective evidence suggests that PBM has a meaningful analgesic effect compared to placebo or conventional therapies. Still, the strength and consistency of these outcomes varied, depending on the clinical condition, study design, and assessment tools used.

Many of the trials reported both statistically and clinically significant reductions in pain levels, measured using validated instruments such as the Visual Analogue Scale (VAS), Numerical Rating Scale (NRS), Brief Pain Inventory (BPI), and neuropathic pain questionnaires. In several cases, the degree of pain reduction surpassed the commonly accepted threshold for clinical relevance—typically a drop of more than two points on the VAS (Farrar et al., 2001; Mease et al., 2011). For example, Campos et al. (2024) noted significant improvements in orofacial pain and tension-type headaches following COVID-19; da Silva et al. (2018) observed better pain management in fibromyalgia; Navarro-Ledesma et al. (2022) reported decreased NPRS scores compared to placebo; and Leal et al. (2022) found notable relief in patients with diabetic neuropathy. These outcomes highlight PBM’s therapeutic potential, though the lack of standardized treatment parameters remains a limiting factor for broader clinical application.

Although the heterogeneity of photobiomodulation protocols is widely acknowledged, a more structured comparative analysis of the applied parameters enhances the clinical interpretability of the findings. Overall, the included studies predominantly employed wavelengths within the red and near-infrared spectrum, ranging from 660 to 905 nm, a range associated with greater tissue penetration and modulation of inflammatory and neurophysiological processes (Chow et al., 2009; Hamblin, 2017; Chung et al., 2012; Bjordal et al., 2006). Energy doses varied substantially, from localized applications as low as 4 J/cm^2^ to more intensive protocols exceeding 100 J/cm^2^, reflecting differences in device characteristics, treated surface area, and therapeutic objectives (Hamblin, 2017; Chung et al., 2012; Teixeira et al., 2022). Regarding treatment frequency, most trials adopted two to three sessions per week over periods ranging from three days to twelve weeks, suggesting that repeated and cumulative protocols are more commonly associated with sustained analgesic effects (Navarro-Ledesma et al., 2024; Chow et al., 2009; Vassão et al., 2024; Navarro-Ledesma et al., 2022). Although this methodological diversity limits direct comparisons across studies, organizing stimulation parameters into defined ranges allows the identification of clinically relevant trends and may support the development of more standardized protocols in future investigations.

The repeated observation of positive outcomes across different disorders—such as fibromyalgia, diabetic neuropathy, and chemotherapy-induced neuropathy—points to the likelihood that PBM operates through shared mechanisms of pain modulation, rather than disease-specific pathways. In the context of fibromyalgia, for instance, studies frequently reported not only pain relief but also increased pressure pain thresholds and improvements in overall quality of life (Navarro-Ledesma et al., 2024; Ribeiro et al., 2023; Navarro-Ledesma et al., 2022; da Silva et al., 2018). Comparable trends were seen in diabetic neuropathy (Leal et al., 2022; André Timóteo et al., 2024) and in neuropathic pain resulting from chemotherapy (Teng et al., 2022; Argenta et al., 2017), reinforcing the idea that both central and peripheral modulation of pain pathways may be involved.

Considering the inclusion of heterogeneous chronic pain populations, the consistency of the evidence varied according to the clinical condition evaluated. Overall, findings were more consistent in populations with fibromyalgia and peripheral neuropathic pain—such as diabetic neuropathy and chemotherapy-induced peripheral neuropathy—where multiple trials reported statistically and clinically significant pain reductions compared with control groups (Ribeiro et al., 2023; Teng et al., 2022; Argenta et al., 2017; Navarro-Ledesma et al., 2022; da Silva et al., 2018; Leal et al., 2022; André Timóteo et al., 2024). In contrast, results were more variable in specific musculoskeletal conditions, including temporomandibular disorders and chronic neck pain, in which some studies demonstrated significant within-group improvements without clear between-group differences when compared with active or placebo comparators (Brochado et al., 2018; Aisaiti et al., 2021; Teixeira et al., 2022; Bonazza et al., 2025). This variability may reflect differences in underlying pathophysiological mechanisms, outcome selection, and the heterogeneity of photobiomodulation protocols employed across studies. Distinguishing these condition-specific patterns supports a more balanced interpretation of the evidence and highlights the clinical contexts in which photobiomodulation appears to have greater therapeutic potential.

In addition to its analgesic properties, PBM was associated with functional and psychosocial benefits in several studies. Campos et al. (2024) documented improvements in sleep quality and daily activities, as measured by the functional components of the BPI. Likewise, da Silva et al. (2018) and Navarro-Ledesma et al. (2022) reported enhanced quality of life and greater tolerance to physical activity. These effects suggest that PBM may play a broader role in promoting well-being beyond pain control. However, results were not entirely consistent across studies—for instance, Teixeira et al. (2022) found no significant change in functional outcomes, which could reflect differences in treatment duration, PBM parameters, or sample characteristics.

Outcomes in TMD populations showed greater variability. While Aisaiti et al. (2021) reported notable improvements in joint-related pain, Brochado et al. (2018) found no significant differences between PBM and manual therapy. These inconsistencies may be due to differing methodologies, including dosage, frequency of application, and patient selection criteria. Still, the broader body of evidence suggests that PBM likely influences pain through neurophysiological modulation, including effects on the autonomic nervous system and microVAScular regulation. Even so, the absence of uniform treatment protocols continues to hinder the consolidation of robust, generalizable evidence in this area.

A consistent strength across all included studies was the favorable safety profile of PBM. None of the trials reported serious adverse events or treatment-related participant dropouts. Minor side effects, such as localized warmth or slight discomfort, were infrequent and occurred at similar rates in both active and placebo groups (Al-Quisi et al., 2019). Similar findings have been reported in trials involving musculoskeletal pain and fibromyalgia, where PBM was well tolerated and no severe adverse effects were observed (Chow et al., 2009; Bjordal et al., 2006). This further supports PBM as a safe and accessible option, particularly for individuals with multiple comorbidities or poor response to medication-based treatments.

That said, several methodological limitations must be considered. A major issue was the wide variability in technical parameters across studies. Reported wavelengths ranged from 660 to 905 nm, with power outputs typically between 70 and 100 mW for point-source lasers. Energy doses varied substantially, from as little as 4 J/cm^2^ to over 130 J/cm^2^, while treatment schedules ranged from two to three sessions per week over two to twelve weeks. Devices also differed considerably, including handheld lasers, LED cluster probes, and full-body PBM systems. This heterogeneity complicates cross-trial comparisons and makes it challenging to establish standardized and optimal therapeutic protocols (Hamblin, 2017; Chung et al., 2012).

Second, most trials enrolled relatively small samples—often between 20 and 80 participants—and were conducted in single-center settings, which limits both statistical power and external validity. Moreover, follow-up assessments were generally short, frequently ending within eight weeks after the final treatment session. This lack of long-term data raises important questions about the durability of PBM’s clinical benefits (Chow et al., 2009; Tumilty et al., 2010).

Third, several studies exhibited risk of bias in at least one domain. While most were rated as low risk overall, common issues included insufficient details on randomization (Brochado et al., 2018), lack of reporting on adherence and protocol fidelity (Vassão et al., 2024), and incomplete data on participant retention (Bonazza et al., 2025). Greater methodological transparency is essential for strengthening internal validity in future investigations.

In conclusion, the current evidence base positions photobiomodulation as a promising non-pharmacological intervention for managing chronic pain, particularly in cases of fibromyalgia and peripheral neuropathies. Nonetheless, substantial variability in treatment protocols, outcome measures, and follow-up duration continues to limit the development of standardized clinical guidelines. To advance the field, future RCTs should focus on larger, multicenter cohorts, employ consistent PBM parameters, and include longer follow-up periods to evaluate sustained effects. Furthermore, integrating PBM within multimodal pain management strategies, such as physical therapy, structured exercise, and behavioral interventions, may enhance outcomes and should be explored in greater depth.

## Data Availability

The original contributions presented in the study are included in the article/supplementary material, further inquiries can be directed to the corresponding author.
